# Single Chain Mean-Field Theory Study on Responsive Behavior of Semiflexible Polymer Brush

**DOI:** 10.3390/ma14040778

**Published:** 2021-02-07

**Authors:** Yingli Niu, Xiangyu Bu, Xinghua Zhang

**Affiliations:** School of Science, Beijing Jiaotong University, Beijing 100044, China; ylniu@bjtu.edu.cn

**Keywords:** computational methods, polymers, SCMFT, MPI

## Abstract

The application of single chain mean-field theory (SCMFT) on semiflexible chain brushes is reviewed. The worm-like chain (WLC) model is the best mode of semiflexible chain that can continuously recover to the rigid rod model and Gaussian chain (GC) model in rigid and flexible limits, respectively. Compared with the commonly used GC model, SCMFT is more applicable to the WLC model because the algorithmic complexity of the WLC model is much higher than that of the GC model in self-consistent field theory (SCFT). On the contrary, the algorithmic complexity of both models in SCMFT are comparable. In SCMFT, the ensemble average of quantities is obtained by sampling the conformations of a single chain or multi-chains in the external auxiliary field instead of solving the modified diffuse equation (MDE) in SCFT. The precision of this calculation is controlled by the number of bonds $N_{m}$ used to discretize the chain contour length *L* and the number of conformations *M* used in the ensemble average. The latter factor can be well controlled by metropolis Monte Carlo simulation. This approach can be easily generalized to solve problems with complex boundary conditions or in high-dimensional systems, which were once nightmares when solving MDEs in SCFT. Moreover, the calculations in SCMFT mainly relate to the assemble averages of chain conformations, for which a portion of conformations can be performed parallel on different computing cores using a message-passing interface (MPI).

## 1. Introduction

Advances in studies of natural and artificial polymer systems in both bioscience and material science continually raise new challenges for theories in polymer physics. In bioscience, biomacromolecules and some subcellular structures in organisms, such as RNA, DNA, proteins, actin filaments, and microtubules, have semiflexible linear structures that endow their physiological functions [1,2]. In material science, functional polymers such as liquid crystal polymers and conjugated polymers are typical semiflexible polymer chains that play an important role in the development of next-generation display devices and photovoltaic cells [3]. Moreover, the development of microelectronic technology has encountered bottlenecks due to the resolution limit in classic lithography machines. A new generation of photolithography technology using block copolymer self-assembled nanostructures requires low-molecular-weight polymers [4]. As the molecular weight of polymers is sufficiently low, namely, the ratio of length of the chain backbone to the Kuhn length is decreased, the flexibility of the chain is decreased. Generally, all of these systems have the common characteristics of a self-assembled structure formed by the semiflexible chains, exhibiting an anisotropic and length-scale dependence structure and a response behavior under the action of an external field.

Research on the conformation statistics semiflexible polymer model and the development of methods for both theory and simulation are still the most pressing frontier [5]. The best chain model that can describe the characteristics of chain flexibility, the degree of freedom of orientation, and the multi-scale is the worm-like chain (WLC) model. It can describe the statistical properties from the microscopic persistence length $l_{p}$ to the mesoscopic length of the backbone contour length of a chain *L*. Therefore, it is suitable for research on the multi-scale problem. In contrast, the commonly used Gaussian chain (GC) model is only applicable around the scale of the radius of gyration $R_{g}$. If one ignores the short wavelength modes, formulation of the modified diffuse equation (MDE) of WLC recovers that of the GC model [6]. In addition, the WLC model depends on the degree of freedom of the bonds’ orientation. Therefore, it can describe a system with orintational order, e.g., the liquid crystal. Furthermore, the WLC model can be used to study the chain flexibility effects, i.e., from the limit of a completely rigid polymer, L/a→0, to the limit of fully flexible Gaussian chain L/a→∞.

In self-consistent field theory (SCFT), the statistical properties of the WLC model are described by the end integral propagator q(r,u,s) (distribution function), which satisfies the modified diffusion equation (MDE):

(1) $$\frac{\partial }{\partial s}q\left(r,u,s\right)=\left[\frac{1}{2l_{p}}\nabla_{u}^{2}-u\cdot \nabla_{r}-\omega \left(r,u\right)\right]q\left(r,u,s\right)$$

with the initial condition of q(r,u,0)=1. This is a partial differential equation (PDE) in the space of a three-dimensional positional coordinate r, a two-dimensional angular coordinate u, and a one-dimensional coordinate of the contour coordinate *s* along the backbone of the chain. Additionally, the coupling of multi-degrees of freedom and the coupling of the multi-scale of a chain increase the solving difficulty. The solving difficulty restricts the development of theoretical studies on semiflexible polymer systems. Since the 1970s, researchers have made unremitting efforts. Yamakawa et al. used graphs to represent moments in the Fourier–Laplace space and solved the propagator by redefining the summation rules [7]. Spakowitz and Wang et al. attributed yamakawa’s graph method to a one-dimensional random walk problem and expressed the propagator with a simple form of continued fraction [8]. Stepanow obtained a matrix expression equivalent to the continuous fraction by solving the propagator from the Dyson equation [9]. Chen and Liu et al. solved Equation (1) under a weak inhomogeneous approximation and gave a Landau-type expression of free energy [10,11]. Morse and Fredrickson et al. adopted the ground state approximation and used a finite difference scheme for positional and orientational spaces. This work showed a chain orientation distribution on the interface that could not be described by the GC model [12]. Tang and Yang et al. gave an efficient difference method in orientational space based on the finite element algorithm [13]. In view of using the Gaussian chain model for success, Fredrickson et al. suggested using the pseudopotential spectrum method to solve Equation (1) [14]. Jiang et al. applied this method to study the interface between the isotropic phase and the nematic liquid crystal phase [15]. Recently, Tang and Yang studied the two-dimensional self-assembly structure of semiflexible diblock copolymers using the pseudopotential spectrum method [16]. Jiang and Chen solved an MDE of the WLC model in a three-dimensional positional space [17] and studied the influence of flexibility on the phase behavior of WLC diblock copolymers.

Although the theoretical methods of semiflexible polymers developed rapidly in recent years, only low-dimensional ordered structures with higher symmetry were studied to avoid the difficulty with multi-degrees of freedom [18]. Moreover, it is hard for the existing theoretical methods to solve the confinement effects on semiflexible polymers, especially in the vicinity of curved surfaces. First, symmetry of the confinement system is low, and as a consequence, it cannot avoid a time-consuming solution in high-dimensional MDE. Secondly, giving the boundary condition satisfied by the propagator *q* due to the confinement surface is not trivial. This boundary condition relates to the curvature of the surface and to the relative orientation of the polymer and the surface [19,20]. Finally, the propagator *q* of the WLC model is highly correlation with the contour variable, *s*, due to chain flexibility. As the chain becomes rigid, the rigidity of Equation (1) also becomes stronger at the same time from the point view of numerical mathematics. The solution requires a more reliable scheme to ensure the stability and accuracy of the algorithm [15]. The difficulties mentioned above are the fundamental reasons why the thermodynamic properties of semiflexible polymer systems near the surface have not been systematically studied. An efficient theoretical method is demanded for the studied of a semiflexible polymer system. Recently, we used single chain mean-field theory (SCMFT) in the study of semiflexible polymer surface and interface problems [6]. The SCMFT was discussed by early works, such as Ben-Shaul et al. and Carignano et al., who proposed the concept of stochastic sampling of chain conformations in self consistent field theory schemes [21,22], Müller et al. proposed the concept of partial enumeration schemes [23,24], and Bonet Avalos et al. and Zaid et al. explicitly proposed the concept of single chain mean-field theory [25,26]. In this theory, the Monte Carlo method is used instead of the numerical solution of Equation (1) to obtain the ensemble average in the auxiliary field. SCMFT is superior in solving semiflexible polymer brushes.

## 2. Single Chain in Mean-Field Theory

It is worth noting that the orientational and positional degrees of freedom of the WLC monomer are not independent from each other. According to the definition of the WLC model, the configuration of the chain is a smooth inextensible spacial curve. This condition is ensured by introducing the following constraint into the configuration integral in the partition function:

(2) $$\delta \left[u\left(s\right)-\frac{dr(s)}{ds}\right]$$

where δ[|u(s)|−1]. Alternatively, this constraint can also be expressed as a global constraint δ[∫u(s)ds/L−1].

Therefore, the orientational and positional freedom are correlated with each other. Either the position {r(s)} of the monomer or orientation {u(s)} of the bond alone can comprehensively describe the conformation of the polymer. According to this consideration, the propagator *q* used in SCFT depending on both {r(s)} and {u(s)} is an excessive description. In fact, the major difficulty in solving Equation (1) is the coupling between orientation and position degrees of freedom. Alternatively, solving the integral equation does not have this difficulty. Generally, from the perspective of applied mathematics, the PDE can always be attributed to a diffusion problem under a given constraint and can be expressed by a path integral form. For example, the Schrödinger equation satisfied by the wave function can be replaced by a Feynman path integration in quantum mechanics. According to this consideration, the ensemble average of any quantity can be computed by the path integral instead of solving the equation of propagator. The diffusion path in the mean field can be sampled using the Monte Carlo method. Here, we formulate the method briefly using the polymer brush as an example.

Consider *n* semiflexible chains with a total backbone contour length *L*, persistence length $l_{p}$, and excluded diameter *d*, which are grafted on a planar impenetrable surface. The configuration of the chain is described by a spacial cure R(s), where s∈[0,L] is the contour variable. By defining the effective Kuhn length $a\equiv 2l_{p}$, we can introduce an effective polymerization N=L/a. The Hamiltonian of a conformation is

(3) $$\begin{array}{c} \beta H_{0}\left[u\left(s\right)\right]=\frac{l_{p}}{2}\int_{0}^{L}ds{\left|\frac{du(s)}{ds}\right|}^{2}. \end{array}$$

By re-scaling the contour variable with s=s/L, we have

(4) $$\begin{array}{c} \beta H_{0}\left[u\left(s\right)\right]=\frac{l_{p}}{2L}\int_{0}^{1}ds{\left|\frac{\partial u(s)}{\partial s}\right|}^{2}=\frac{1}{4N}\int_{0}^{1}ds{\left|\frac{\partial u(s)}{\partial s}\right|}^{2}. \end{array}$$

The distribution function of a single conformation is

(5) $$\begin{array}{c} P_{0}\left[R\left(s\right),u\left(s\right)\right]=\exp\left\{-\beta H_{0}\left[u\left(s\right)\right]\right\}\prod_{s}\left\{\delta \left[|u\left(s\right)|-1\right]\delta \left(u-\frac{\partial R(s)}{\partial s}\right)\right\}\delta \left[R\left(0\right)-R_{d}\right]. \end{array}$$

The first two δ functions implicate that the position of segments, R(s), and the tangent vector, u(s), are not independent from each other due to the constraint of inextensibility, which leads to an extra difficulty in solving this mode with MDE or Dyson equations, compared with the Gaussian chain mode. The last δ function means that one end of the polymer is grafted on a surface at position $R_{d}$.

The microscopic contour-averaged density operators are defined as

(6) $$\begin{array}{c} \hat{\rho }\left(r,u\right)=N\sum_{i=1}^{n}\int_{0}^{1}ds\delta \left[r-R_{i}\left(s\right)\right]\delta \left[u-u_{i}\left(s\right)\right]. \end{array}$$

Typically, the excluded volume interaction between two bonds with orientations of u and $u^{\prime }$ has the following Onsager’s form:

(7) $$\begin{array}{c} \beta V\left(r,r^{\prime },u,u^{\prime }\right)=2da^{2}\delta \left(r-r^{\prime }\right)\left|u\times u^{\prime }\right|; \end{array}$$

The interaction between the monomer and the grafting surface is described by the following potential:

(8) $$\begin{array}{c} \beta H_{I}\left(r\right)=\left\{\begin{array}{cc} 0 & z>0\\ \infty & z\leq 0 \end{array}\right., \end{array}$$

which means the surface is impenetrable. The partition function can be written as a function with respect to the auxiliary field ω:

(9) $$\begin{array}{c} Z=\int D\left\{\omega \right\}\exp\left\{-\beta F[\omega ]\right\} \end{array}$$

where a Hubbard–Stratonovich transformation is used in the derivation; the effective Hamiltonian is defined as

(10) $$\begin{array}{c} \beta F\left[\omega \right]\equiv \frac{1}{2}\int drdudu^{\prime }\frac{\omega \left(r,u\right)\omega \left(r,u^{\prime }\right)}{2da^{2}\left|u\times u^{\prime }\right|}-n\ln Q\left[i\omega \right], \end{array}$$

and

(11) $$\begin{array}{ccc} Q[i\omega ] & \equiv & \int D\left\{R\left(s\right),u\left(s\right)\right\}\prod_{s}\left\{\delta \left[|u\left(s\right)|-1\right]\delta \left[u-\frac{\partial R(s)}{\partial s}\right]\right\}\delta \left[R\left(0\right)-R_{d}\right]\\ & & \times \exp\left\{-\beta H\left[u\left(s\right)\right]-i\int ds\omega \left[r\left(s\right),u\left(s\right)\right]-\beta \int drduH_{I}\left(r\right)\hat{\rho }\left(r,u\right)\right\}, \end{array}$$

is the single chain partition function. Considering the mean-field approximation δF/δiω=0, self-consistent field equations can be obtained:

(12) $$\begin{array}{ccc} i\omega (r,u^{\prime }) & = & 2da^{2}\int du^{\prime }\left|u\times u^{\prime }\right|\rho \left(r,u^{\prime }\right), \end{array}$$

where

(13) $$\begin{array}{ccc} \rho (r,u) & \equiv & \langle \hat{\rho }\left(r,u\right)\rangle =\frac{A\sigma }{Q}\frac{\delta Q}{\delta i\omega }\\ & = & \frac{A\sigma }{Q}\int D\left\{R\left(s\right),u\left(s\right)\right\}\prod_{s}\left\{\delta \left[|u\left(s\right)|-1\right]\delta \left[u-\frac{\partial R(s)}{\partial s}\right]\right\} \end{array}$$

(14) $$\begin{array}{cc} & \times N\int_{0}^{1}ds\delta \left[r-R\left(s\right)\right]\delta \left[u-u\left(s\right)\right]\exp\left\{-\beta H\left[u\left(s\right)\right]-iN\int_{0}^{1}ds\omega \left[r\left(s\right),u\left(s\right)\right]\right\} \end{array}$$

where σ is the grafting density and *A* is the area of the grafting surface.

In SCFT, this ensemble average is computed using the propagator *q*, which is difficult to obtain. To overcome this problem, the ensemble average is computed directly by the path integral. Plenty of conformations of the chain in the auxiliary field are sampled by Monte Carlo simulation. In the simulation, the WLC chain is discretized as a path with $N_{m}$ discrete steps. The *i*th bonds of the *j*th conformation is characterized by the position $r_{i}^{j}\left(x_{i}^{j},y_{i}^{j},z_{i}^{j}\right)$ and by orientation $u_{i}^{j}\left(\theta_{i}^{j},\varphi_{i}^{j}\right)$. By considering s/L→s and $L=2l_{p}N$, the Hamiltonian becomes

(15) $$\begin{array}{c} \beta H_{0}=\epsilon \sum_{i=1}^{N_{m}-1}{\left|u_{i+1}-u_{i}\right|}^{2}, \end{array}$$

where $\epsilon =N_{m}/4N$ characterizes the flexibility of the chain. As $N_{m}\to \infty$, the continuous WLC recovers. *M* most probable paths in auxiliary field ω(r,u) are sampled by the metropolis method with an acceptance criterion of P=min{1,exp(−βΔE)} for ensemble averages where

(16) $$\begin{array}{c} \beta \Delta E=\epsilon \sum_{i=1}^{N_{m}-1}\left(|u_{i+1}^{j+1}-u_{i}^{j+1}{|}^{2}-{\left|u_{i+1}^{j}-u_{i}^{j}\right|}^{2}\right)+\frac{N}{N_{m}}\sum_{i}^{N_{m}}\left\{\omega \left[R_{i}^{j+1},u_{i}^{j+1}\right]-\omega \left[R_{i}^{j},u_{i}^{j}\right]\right\} \end{array}$$

is the energy change of the (j+1)th trial move. Then, the ensemble average of any operator $\langle \hat{A}\left[u_{i}\right]\rangle$ depending on the chain conformations can be calculated. For example, the density distribution profile can be calculated by

(17) $$\begin{array}{c} \rho \left(r,u\right)=\frac{\sigma A}{M}\sum_{j=1}^{M}\frac{N}{N_{m}}\sum_{i=1}^{N_{m}}\delta \left(r-r_{i}^{j}\right)\delta \left(u-u_{i}^{j}\right). \end{array}$$

In order to generate the independent conformations efficiently, the pivot Monte Carlo trial move is considered in SCMFT. The partial chain between the random chosen monomer and the free end is rotated at a random angle around a random chosen axis, according to

(18) $$r^{\prime }=n\left(n\cdot r\right)+\left[r-n\left(n\cdot r\right)\right]\cos\theta +\left(n\times r\right)\sin\theta ,$$

where r and $r^{\prime }$ are the vectors before and after rotation, respectively; θ is the rotating angle; and n is the random chosen axis.

## 3. Advantages of WLC SCMFT

The major difference between SCMFT and SCFT is the method used to compute the ensemble average. In SCFMT, the chain conformations are sampled to compute the ensemble average, while in SCFT, the distribution function, *q*, is used. Typically, the mean-field theory computation is used to study the inhomogeneous polymer system, which can be considered the effect of the constraint, for example, the boundary conditions or the conformation constraints on the chain. In SCMFT, the constraints can be directly applied on the Monte Carlo trial moves during the sampling process. However, in SCFT, the constraints are imposed on the distribution function as the boundary condition or the initial condition, which leads to discontinuity in the PDE. The polymer brush is a typical example of the polymer interfacial system. In this system, the chain ends are grafted on the surfaces. This constraint imposes a δ function as the initial condition in the MDE, which brings about the discontinuity. In order to deal with this problem, generally, an broadening, $\lim_{\sigma \to 0}\frac{1}{{\left(\sqrt{2}\sigma \right)}^{3}}\exp\left(-\frac{|R-R_{d}{|}^{2}}{2\sigma^{2}}\right)=\delta \left(R-R_{d}\right)$ is considered, which introduces an artificial length scale, σ. WLC is a multi-scale model, and any extra length would affect its statistical behavior [27]. As a consequence, much more discrete grids are needed in the positional space. The ensemble average in SCMFT is based on the path integral where the integral equations are involved. The grafting point constraint is equivalent to introducing a δ function in an integral, which equals to 1. In practice, one only needs to fix the position of one chain end on the grafting point during Monte Carlo sampling. Therefore, SCMFT is superior in describing the constraints.

SCMFT is very efficiency for the WLC system with low symmetry. In SCFT, the WLC is considered a diffusion in five-dimensional space (the {r,u} space). This high-degree-of-freedom problem is hard to be solved from the viewpoint of a numerical scheme development. Therefore, almost all of the published work on WLCs deal with the system with some symmetry [18]. A dimensionality reduction can be made according to the symmetry. In SCMFT, only the degrees of freedom of the monomer position r(s) are considered and the orientations of the bonds can be computed according to Equation (2). Then, the computation is a diffusion in the positional space and the Monte Carlo sampling is always performed in the three-dimensional space, which is more efficient than that of SCFT. If the system with some symmetry is considered, the conformations R(s) in three-dimensional space are projected onto inhomogeneous directions to compute the ensemble average of field ρ and ω.

Usually, to accelerate the computation of SCFT, a parallel algorithm is used. However, the performance is limited by the algorithm solving the MDE. Generally, the self-consistent iteration process cannot be vectorized. In SCFT, the parallel algorithm is only used to accelerate fast Fourier transition (FFT) when solving an MDE using the pseudo spectra method [14]. In this method, the simulation box is sliced into slabs along one direction. The FFT computations within these slabs are distributed to different computing cores. The parallel scale is limited by the number of slabs. In practice, the number of computing cores is much less than that of the discretized grids in this direction. To enlarge the scale of the parallel algorithm, the simulation box can be further sliced in two directions or in all three directions. Nevertheless, the parallel scale is limited. Moreover, the efficiency of parallel computing is determined by the frequency and scale of the communication between different cores. In the parallel algorithm of SCFT, the computations on different cores are highly correlated. The communication is needed every evolution step along *s* when solving an MDE.

SCMFT is suitable for acceleration using the parallel algorithm. The major task in computing the ensemble average in SCMFT is to generate a sufficiently large number of uncorrelated conformations in the same auxiliary field. This large-scale sampling process can be divided into a large number of independent sub-ensembles, and the sampling in these sub-ensembles can be assigned to different cores for parallel computing, which can reach thousands. In fact, the parallel algorithm of SCMFT corresponds to slicing in the time dimension. For an equilibrium system, more time slicing is better. In practice, this algorithm is performed using a message-passing interface (MPI). Certainly, it can also be performed on a GPU platform. These sub-ensembles are completely independent for a given auxiliary field. Communication only takes place when the auxiliary field is updated according to the iteration algorithm, which is scarce compared with the parallel algorithm of SCFT. Therefore, SCMFT has high parallel efficiency naturally due to the conformation-based ensemble average.

From the viewpoint of saving memory during the computation, in SCMFT, only the conformation of the chain is frequently operated. It is an array storing the position of the monomers along the chain that contains $3N_{m}$ floating numbers. Here, $N_{m}$ is the numbers of discretized points of contour variable *s*. However, SCFT is a memory-consuming method. In SCFT, the propagator is the frequently operated object. It requires a quite large array to store the probability of all the grids in the {r,u} space for all contour variables *s*. It contains $N_{m}\times N_{x}^{3}\times N_{\theta }\times N_{\varphi }$ floating numbers, where $N_{x}$ is the grids discretized in one direction of positional space, and $N_{\theta }$ and $N_{\varphi }$ are the grids discretized in the orientational space. The low memory demand leads to the high efficiency within a single computing core and the high efficiency of communication between the parallel computing cores.

One more advantage of SCMFT is that it is suitable for demonstrating the relation between the structure and the conformations. Generally, the structure of the system is determined by the conformations of the chains, and in SCMFT, the conformations of the chain are explicit. According to these requirements, the typical conformations of the chains in a given constraint can be sampled. Then, the conformations can be used to interpret the structure intuitively. However, the SCFT cannot provide a direct description of the conformations. To investigate the conformation state, it needs the complex definitions of the multi-point correlation function, which involves computation. For example, to discuss the conformation of the chains adsorbed on the nanoparticles, the tail, loop, and bridge conformations are computed by decomposition into adsorbed and free propagators [28,29]. Alternatively, one also can use Monte Carlo simulation to sample the typical conformations in the auxiliary field computed from SCFT to make up the shortcoming of the SCFT.

## 4. Application on Polymer Brushes

Polymer brushes are the typical technology for surface modification. In these system, the chains are grafted on the substrate, which introduces a constraint on the degree of freedom of the polymer end. These constraints lead to discontinuity in the theory, which is hard to solve in SCFT. WLC-SCMFT is useful for the study of polymer brushes. Here, five examples of the WLC brushes grafted on both planar and curved surfaces are demonstrated.

### 4.1. Nematic Brush

**Figure 1:**
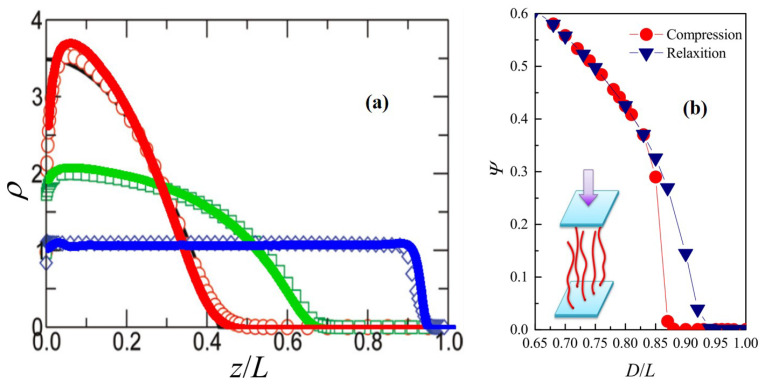
(**a**) The single chain mean-field theory (SCMFT) results (lines) were compared with the results from self-consistent field theory (SCFT) (scatters), which were from the reference. These results were computed with the same chain flexibility L/a=30 but different grafting densities 2daσ=0.25 (red), 2.0 (green) and 35.0 (blue). (**b**) The inset shows the scheme diagram of compression of the nematic brushes. A tilting transition takes places when the nematic brushes are subjected to the external compression. A hysteresis loop of the tilting transition during the compression and relaxation process indicates that this is a first-order transition. Reprinted with permission from ref. [6]. Copyright 2015 AIP Publishing.

Firstly, the polymer brush on a planar surface was considered. Here, the WLC was discretized into $N_{m}=100$ monomers, and the flexibility L/a=30 was adopted, which is the same as used in the SCFT work [30]. In this example, the chain was flexible and their conformations were determined by the effective grafting density 2daσ. In the low grafting condition, the prediction from the GC model recovered with a narrow depletion zone, a parabolic profile, and an exponential tail. In the high grafting condition, the high stretched limit recovered with a step function-like profile. The results of SCMFT (lines) and SCFT (scatters from Reference [30]) are compared in Figure 1. This is a lateral homogeneous system with the density distribution only depending on *z* in the positional space. Therefore, a single chain was simulated in the SCMFT computation. Typically, $10^{7}$ conformations are sampled for an ensemble average. It can be observed that the results from SCFT and SCMFT are consistent with each other, especially for the dense grafting condition, e.g., 2daσ=35.0. There are deviations for the bush with a low grafting density at 2daσ=0.25. For the dense grafting condition, the chains in the brush were highly stretched and the density profile was similar to the step function around z/L≈1, which needs to dramatically increase the grids in the positional and orientational spaces to obtain the computing accuracy of propagators in SCFT. On the contrary, in SCMFT, the explicit stochastic quantities are the chain conformations subject to the auxiliary field, which can be well sampled by the Monte Carlo simulation. The difficulty in using SCMFT is from the condition with a low grafting density, where the chains take coil conformations. According to the center limited theorem, when $N_{m}\to \infty$, the random walk behavior recovers. To obtain the results of SCFT for the low grafting condition, a large $N_{m}$ is needed in SCMFT. In practice, $N_{m}∼10^{3}$ is sufficient.

For the high grafting brush or the rigid chain limit, conformation of the grafting chains becomes anisotropic and forms a nematic order. The responsive behaviors of the nematic brush are quite different from that of the isotropic brush and depend on the chain flexibility. Using SCMFT, the responsive behaviors of a nematic brush to uniaxial compression can be studied [6]. Under the compression, lateral symmetry breaking takes place for the semiflexible chain condition. In this condition, the nematic order is retained but the nematic axis tilts to an angle relative to the compression direction. The order parameter of the breaking of rotational symmetry can be defined as $\psi =\langle 2\int_{0}^{1}ds\cos^{2}\varphi \left(s\right)-1\rangle ,$ where φ is the preference tilting angle in the horizontal plane. This behavior is similar to the smectic A to smectic C transition. As shown in Figure 1b, the order parameter ψ shows a hysteresis loop with respect to the compression and relaxation process, which means this tilting transition is a first-order transition.

### 4.2. Azo-Polymer Brush

Introducing new length scales or new degrees of freedom modified by the molecular structure is a basic way to design new properties and structures of a polymer system. The azobenzene compound is a molecule that has two isomers, i.e., the trans- and the cis-conformations. The trans-state is the thermally stable conformation. Upon ultraviolet (UV) light irradiation, the azobenzene transititions to the cis-state. Adding an azo bond on the polymer brush introduces the light-controlled degree of freedom that brings new responsive mechanisms of the brush. This ability to reversibly photoswitch between isomers enables azobenzene to drive the tail of the grafted chains. This can be used to design novel structures with fancy properties [31]. In theory, the difficulty is in describing the azo polymer from the angle constraint of the azo bond $\delta (\gamma_{azo}-\alpha )$, where α=π or π/3 represents the bond angle of the trans or cis isomers, respectively. As the constraint of the grafting point in positional space, this bond angle constraint introduces discontinuity into the orientational space, which is difficult to deal with in SCFT. As a consequence, the theoretical work on azo polymer using SCFT has not been reported.

**Figure 2:**
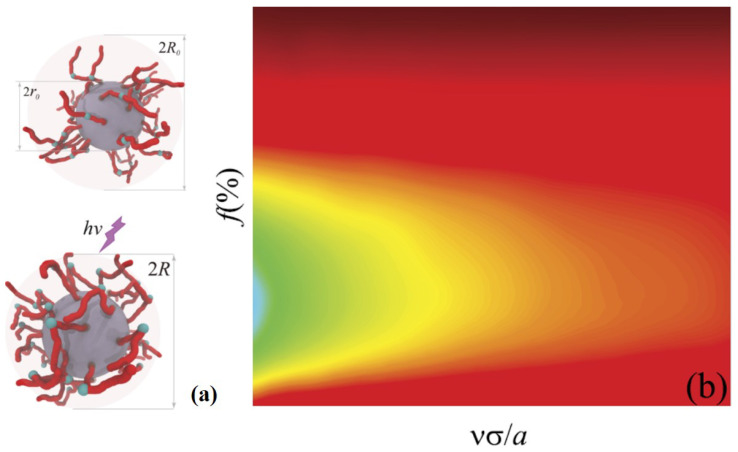
(**a**) Scheme diagram of the azo polymer-coated spherical nanoparticle. (**b**) The light-operating performance of the size shrinking of the nanoparticle depending on chain grafting density and *f* of the semiflexible chain condition. Reprinted with permission from ref. [32]. Copyright 2017 AIP Publishing.

Taking the advantages of SCMFT, the properties of the azo polymer brushes can be well described. As an example, a light-responsive spherical nanoparticle grafted with azo polymer brushes was studied theoretically. As the result of the UV light induced trans-cis isomer switching of the azo group, such a core-shell nanoparticle was subjected to contraction in terms of its overall size upon UV light irradiation [32], as shown in Figure 2a. The assembly of these smart nanoparticles set a playground to study phase transitions, including sol-gel [33] and glass transition [34], because the volume fraction of particles, which triggers these phase transitions, can be conveniently manipulated by light. Immersing these nanoparticles in a liquid crystal solution, photoswitching of the azo polymer configuration can orient the surrounding liquid crystal molecules, leading to different types of topological defects around the nanoparticle [35]. The performance of this mechanism can be characterized by the brush height ratio between before and after UV irradiation, $P_{1}$. It is determined by the competition of length scales of the tail and the whole chain and can be optimized using the relative position of an azo bond along the chain *f*. The partial chain with s<f and s>f are called the tail and spacer, respectively. A large *f*, i.e., a long spacer, ensures that the orientation of the tail cannot be affected by the substrate. A small *f*, i.e., a long tail, means a large chain can be operated. Moreover, both intrinsic properties of polymer brushes, grafting density and chain flexibility, affect the performance. A brush without UV requires high grafted density and flexibility of the chain to extend the chains. Upon UV irradiation, the brush must be sufficiently loose to hide the brush tail and the chain must be sufficiently rigid such that the tail can be controlled by the azo bond. Figure 2b demonstrates the dependence of $P_{1}$ on both *f* and grafting density νσ/a of the semiflexible chain condition as an example. There is an optimized *f* for any given grafting density.

### 4.3. End Reactive Polymer Brushes

**Figure 3:**
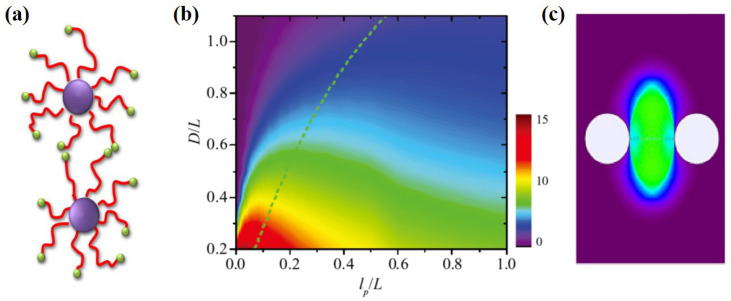
(**a**) Scheme diagram of the interaction between two spherical particles coated with a reactive end polymer. (**b**) The reaction parameter $P_{2}$ as the function of *D* and $l_{p}$. (**c**) The overlapping region of polymer ends from two particles in the semiflexible chain condition. Reprinted with permission from ref. [37]. Copyright 2017 Wiley-VCH Verlag GmbH & Co. KGaA, Weinheim, Germany.

End reactive polymer brushes can be used to construct the structurally dynamic material, which is a potential technology for self-healing materials [36]. Optimizing reactivity and thereby improving the self-healing ability at the most fundamental level poses an urgent issue for the application of such materials. The minimal model of this system is to compute an effective interaction between two nanoparticles coated with end reactive polymer brushes, as shown in Figure 3a. For brush systems without lateral homogeneity, the auxiliary field on the chains are different from each other. In this condition, the chains should be treated explicitly on the surface. It is formidable task to solve the present system in the formation of SCFT owing to the difficulty of treating the boundary condition on the surface of nanoparticles and of developing high-performance numerical algorithms adapted to all $l_{p}$. Alternatively, in SCMFT, the ensemble average of any operator can be obtained by the Monte Carlo simulation of the nanoparticles grafted with *n* ideal chains in the mean field. To treat the grafted points explicitly, the surface of a sphere is discretized with polygon. The apexes of the polygon are chosen as the grafting points. SCFMT was used to reveal how chain stiffness regulates the reactivity of dynamic polymers grafted on nanoparticles [37]. The reacting rate can be determined from the product of average end-segment probabilities for chains from two opposing nanoparticles, $P_{2}=\int dr\phi_{\mathrm{end},I}\phi_{\mathrm{end},\mathrm{II}}$, which characterizes the degree of overlap between the chain-end distribution of the two opposing nanoparticles. Here, $\phi_{\mathrm{end},I}$ and $\phi_{\mathrm{end},\mathrm{II}}$ represent the end distribution of polymer brush from *I* and II nanoparticles, respectively. Figure 3b shows the the contour map of the reaction parameter ψ in the plane of *D* and $l_{p}$. The results allow us to identify that semiflexible dynamic polymers possess optimal reactivity and self-healing ability. This can be explained from the point of view of the first-passage time of two trajectories from opposite nanoparticles with the same contour length. Generally, WLC conformations are identical to the trajectories of persistence walk. It has a high efficiency at the reactive end for a search in the space. At the flexible limit, it becomes a random walk, and at the rigid limit, it corresponds to a direct walk. There is a minimum first-passage time for persistence walk for persistence length is comparable with the chain length. However, random walk and direct walk have quite long first-passage times. Figure 3b shows the the typical overlap of chain ends from two opposing nanoparticles for the semiflexible chain condition. The region of possible reactions taking place is distributed into a large area.

### 4.4. Optical Reactive Adhesion

**Figure 4:**
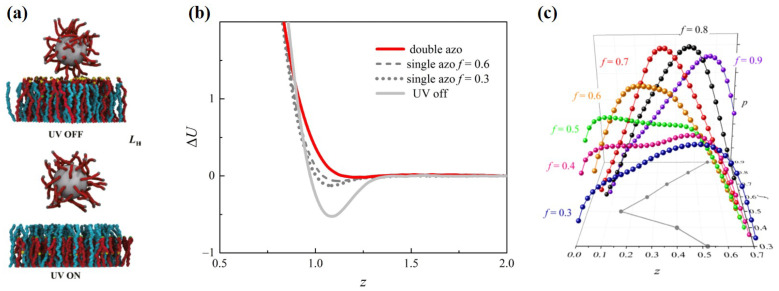
(**a**) Scheme diagram of an optical reactive adhesion smart surface for the adhesion state (above) and erasion state with UV illustration (below). (**b**) Effective interaction between the nanoparticle and the surface with the UV-on and -off states. (**c**) Distribution of receptors on the azo-polymer along the z-axis with different *f*. Reprinted with permission from ref. [39]. Copyright 2019 American Chemical Society.

By virtue of the light-driven mechanism of an azo polymer, an optical reactive adhesion smart surface can be designed as shown in Figure 4a. The spherical nanoparticle is grafted by polymer, which is considered the adsorbate. The end groups of polymer are ligands. The smart surface modified by a hybrid grafted layer is considered the adsorbent, which consists of homopolymers and active polymers with receptors as the end groups. An azo bond is introduced into the active polymer chain of the hybrid polymer brush. Without UV illustration, the receptors are distributed around the outer part of the brush layer and can easily interact with the ligands, which allows the nanoparticle to be adsorbed at the surface. Oppositely, upon UV, the reactive polymers are folded such that the receptors are hidden into the hybrid layer. As a result, an effective interaction between the nanoparticle and the smart surface is decreased, and thus, the nanoparticle is erased from the surface [38]. The performance and mechanism of this azo-polymer-based smart surface were investigated using SCMFT [39]. The adhesion/erasion transition of the system shows that the performance of the smart surface can be characterized by the interaction potential difference between the effective nanoparticle–surface interactions in the UV-on and UV-off states shown in Figure 4b. A critical factor that affects the performance of the smart surface is again the position of the azo-bond along the backbone, *f*, which directly determines the relative length of the tail and the spacer. Upon UV-on, the zo-bond takes the cis-isomer, which makes the tail fold back. However, to erase the nanoparticle efficiently in the UV-on state, the receptors should be embedded as deeply as possible. The effect of *f* on the distribution of the receptor groups in this condition is shown in Figure 4c. The peak of the distribution function first approaches the substrate as *f* decreases and then move away from the substrate for f<0.5. Indeed, the curves of f<0.6 in Figure 4a have typical bimodal characteristics, and thus, each of these distribution curves can be regarded as the superposition of two subtle modes, which is crucial for optimizing the position of the azo-bond along the azo-polymer *f*.

### 4.5. Sliding Polymer Brushes

**Figure 5:**
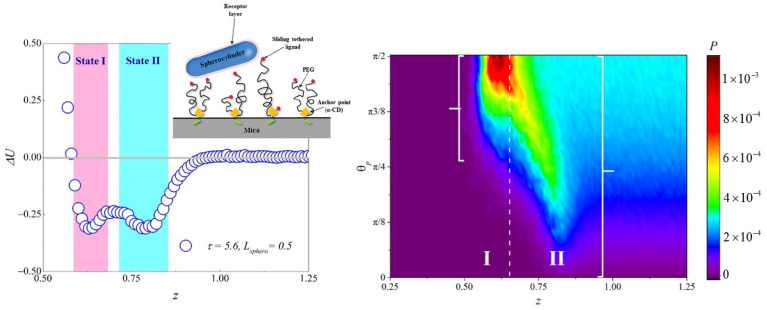
(**a**) Effective adsorption potential of the anisotropic particle and the surface, which shows bistable features: the inset shows a scheme diagram of the system. (**b**) The distribution of the orientational angle and the z axis depending on the positions of anisotropic particle. Reprinted with permission from ref. [41]. Copyright 2019 The Royal Society of Chemistry.

A sliding polymer brush is constructed by adding sliding anchors to the polymer chains, through which the polymer chains can slide with self-regulating lengths. The two ends of the polymer chains are modified by ligands to prevent polymer escape from the anchoring point [40]. The sliding degree of freedom regulates the polymer configuration of the brush and increases the available reaction range, which markedly changes the kinetics and effective interaction potential of the specific binding pairs. This brush introduces a new degree of freedom to the polymer chain, i.e., the repetitive move of the chain through the sliding anchor. It is hard to solve the propagator of the chain in SCFT with the constraint of a sliding anchor. Moreover, the translational degree of freedom of the nanoparticle and the interaction between chain ends and the anisotropic nanoparticle are difficult to formulate in an SCFT model. In SCMFT, these difficulties can be overcome by introducing the corresponding Monte Carlo trial move during sampling. The adhesion of an anisotropic nanoparticle on the sliding brushes was studied using SCMFT [41]. A novel bistable adhesion behavior composed by an energy dominated state (state I) and an entropy dominated state (state II) was found as shown in Figure 5a. This behavior was from the cooperation between the repetitive motion of the sliding polymer and rotational degree of freedom of the spherocylinder. The role of the orientational entropy was quantified by analyzing the distribution of the tilting angle of the spherocylinder, which is shown in Figure 5b. For state I, the orientation angle is around π/2, which indicates that the orientational motion of the particle are largely constrained and lies on the substrate. For state II, the orientation direction is distributed in a wide range of [0,π/2]. Therefore, state II is an orientational entropy dominated state. This system can be used to create a smart surface with a wide sensitive range for bacteria adhesion and a biosensor for experiments and provide a possible explanation for the bistable adhesion behavior of the bacteria on the surface, mediated by type IV pili.

## 5. Summary

In this paper, we reviewed SCMFT for the WLC model, which is based on the path integral description of the ensemble average. In this theory, the ensemble averages of physical quantities can be computed directly by sampling the conformations of the chains. This method is easy to code and is suitable for acceleration using the parallel algorithm. Because it does not involve solving a PDE, it is not necessary to face the difficulty of boundary condition processing and the accuracy and stability of a numerical scheme. Additionally, this method can be generated for systems with all types of polymer models without extra difficulties. Compared with SCFT, the highly stretched condition or rigid chain limit is easy to deal with in SCMFT. However, it is a little difficult to deal with coil conformations, which can be worked out by enlarging the discretized points along the backbone of the chain. Furthermore, the difficulty of WLC is mainly from coupling the degrees of freedom in the orientational and positional spaces. This leads to the high dimensional nature of the MDE in SCFT. However, this coupling indicates that the orientational and positional degrees of freedom are not independent. Therefore, this theory is complicated due to the use of propagators to describe the statistical properties in SCFT. On the contrary, in SCMFT, this problem can be solved naturally from a point view of the conformations. The polymer chains are simulated only in three-dimensional positional space, and the orientations of the bond can be calculated accordingly. This method works well for systems with low symmetry, which involves high dimensional computations and requires large scale memory to store the propagators and the auxiliary field in SCFT. In contrast, in SCMFT, only the three-dimensional conformation is needed for operation and computation.

SCMFT is the best methodology for the study of semiflexible polymers near curved surfaces, a topic of general interest in natural and artificial polymer systems. It provides a clear and comprehensive theoretical picture from both the field and the conformations for a structure with responsive behaviors in the materials. It will be a promising way to optimize the design of the functional polymer optoelectronic devices.

## Data Availability

No new data were created or analyzed in this study. Data sharing is not applicable to this article.

## Abbreviations

The following abbreviations are used in this manuscript:

SCMFT	Single chain mean-field theory
MC	Monte Carlo
MDE	Modified diffuse equation
PDE	Partial differential equation
SCFT	Self-consistent field theory
MPI	Message-passing interface
FFT	Fast Fourier transition
WLC	Worm-like chain
GC	Gaussian chain
